# A narrative review and content analysis of functional and quality of life measures used to evaluate the outcome after TSA: an ICF linking application

**DOI:** 10.1186/s12891-020-03238-w

**Published:** 2020-04-13

**Authors:** Ze Lu, Joy C. MacDermid, Peter Rosenbaum

**Affiliations:** 1 Roth|McFarlane Hand and Upper Limb Centre, St Joseph’s Health Care, London, ON Canada; 2 The School of Rehabilitation Science, McMaster University, Hamilton, ON Canada; 3 Physical Therapy and Surgery, Western University, London, ON Canada; 4 CanChild Centre for Childhood Disability Research, McMaster University, Hamilton, ON Canada

**Keywords:** Patient-reported outcome measures, Total shoulder arthroplasty, ICF, Health-related quality of life, Quality of life

## Abstract

**Background:**

Total shoulder arthroplasty (TSA) is considered as the standard reconstructive surgery for patients suffering from severe shoulder pain and dysfunction caused by arthrosis. Multiple patient-reported outcome measures (PROMs) have been developed and validated that can be used to evaluate TSA outcomes. When selecting an outcome measure both content and psychometric properties must be considered. Most research to date has focused on psychometric properties. Therefore, the current study aims to summarize what PROMs are being used to assess TSA outcomes, to classify the type of measure (International society for quality of life (ISOQOL) using definitions of functioning, disability, and health (FDH), quality of life (QoL) and health-related quality of life (HRQoL)) and to compare the content of these measures by linking them to the International Classification of Functioning, Disability and Health (ICF) framework.

**Methods:**

A literature review was performed in three databases including MEDLINE, EMBASE, and CINAHL to identify PROMs that were used in TSA studies. Meaningful concepts of the identified measures were extracted and linked to the relevant second-level ICF codes using standard linking rules. Outcome measures were classified as being FDH, HRQoL or QoL measures based on the content analysis.

**Result:**

Thirty-five measures were identified across 400 retrieved studies. The most frequently used PROM was the American Shoulder and Elbow Society score accounting for 21% (246) of the total citations, followed by the single item pain-related scale like visual analog scale (17%) and Simple Shoulder Test (12%). Twelve PROMs with 190 individual items fit inclusion criteria for conceptual analysis. Most codes (65%) fell under activity and participation categories. The top 3 most predominant codes were: sensation of pain (b280; 13%), hand and arm use (d445; 13%), recreational activity (d920; 8%). Ten PROMs included in this study were categorized as FDH measures, one as HRQoL measure, and one as unknown.

**Conclusions:**

Our study demonstrated that there is an inconsistency and lack of clarity in conceptual frameworks of identified PROMs. Despite this, common core constructs are evaluated. Decision-making about individual studies or core sets for outcome measurement for TSA would be advanced by considering our results, patient priorities and measurement properties.

## Background

The high prevalence of shoulder pain (7–21%) in the general population results in high, and increasing, levels of disability and health-care costs [1, 2]. Glenohumeral arthritis is the primary cause of shoulder pain and dysfunction in an aging population [1]. Psychological issues such as depression, anxiety, and decreased quality of life (QoL) are associated with chronic musculoskeletal pain [1–4]. The combined physical and psychosocial impacts of shoulder pain are complex and contribute to lower quality of life [2].

Total shoulder arthroplasty (TSA) is reconstructive surgeries that can provide pain relief and restore function in severely damaged arthritic shoulders [5–8]. Such treatments have a predictable outcome for patients with joint destruction arising from pathologies such as osteoarthritis, rheumatoid arthritisand proximal humeral head fracture [8–10]. Previous studies indicated significant improvement of both psychological status and health-related quality of life (HRQoL) by 3-months after surgery [2, 11]. However, implant issues, such as loosened glenoid components can lead to poorer outcomes over the longer-term [3, 7]. While improvement can be expected, normal function cannot be restored and the outcome achieved is variable and dependent on many factors including different surgical indications, soft tissue recovery, subscapularis integrity, and post-operative rehabilitation [5, 12].

To evaluate surgery outcomes, many clinicians and researchers are aware of the importance of measuring pain, functional outcomes, biopsychological health, QoL, and HRQoL [13]. Since the 1990s, numerous patient-reported outcome measures (PROMs) have been developed and validated to assess outcomes in shoulder conditions [14–16]. Researchers have established acceptable levels of reliability, validity, and responsiveness of PROMs such as the American Shoulder and Elbow Society score (ASES) and the Simple Shoulder Test (SST), and synthesized the evidence of psychometric properties in a systematic way [7, 13, 17, 18].

Content validity is a fundamental property of PROM, but relatively unintended to in the literature. Although standard definitions exist for functioning, disability, and health (FDH), rather than HRQoL or QoL [19–21], there is overlap in these concepts and insufficient precision by developers and users with respect to these terms [20]. The absence of a theoretical framework or conceptual definition leads to difficulty of interpreting study results using different outcome measures, since it can be unclear which domains of health are affected by the intervention, and whether differences in outcomes relate to the intervention or the measure [5]. Since few developers provide clear definitions of their latent constructs or how items were mapped to these constructs it is important to do a retrospective evaluation to inform content validation and understand differences in constructs evaluated by commonly used measures.

The World Health Organization (WHO) definitions provided in the International Classification of Functioning, Disability and Health (ICF) and the manual of WHO-Quality of Life brief version (WHOQOL-BREF) [21–24] provided internationally used frameworks, definitions and coding language to describe the impact of health conditions on function, disability and health [21]. With the ICF framework, the concept of FDH refers to the biopsychosocial components and interactions among body structures and function, and activities and participation in the context of the environment and personal factors [25–28]. The QoL is defined by WHO as “a person’s perception of their position in life affected by the culture and value system in which they live and in relation to goals, expectations, standards, and concerns [21, 23].”

However, the terminology HRQoL, remains variably defined by different sources [20]. In HRQoL is specific focus on how QoL is influenced by a health condition [20, 23]. While culture, politics and economic context also affect QoL, those influences are generally not addressed in HRQoL measures or health-related PROM [20]. See Table 1 for orgnizations of the concepts for FDH, QoL, and HRQoL.

**Table 1:** The orginzations of concepts of functioning, diability, and health (FDH), quality of life (QoL), and health-related quality of life (HRQoL)

Concept	Definition adopted	Visual explanation	Item example (name of measurement)
Functioning, disability, and health (FDH)	Biopsychosocial components and interactions among body structures and function, and activities and participation in the context of the environment and personal factors		Is it difficult for you manage toileting? (American Shoulder and Elbow Society Score)
Quality of life(QoL)	A person’s perception of their positions in life affected by the culture and value system in which they live and in relation to goals, expectations, standards, and concerns		How much of a burden do you feel you are on others? (Western Ontario Osteoarthritis Score)
Health-related quality of life (HRQoL)	The subjective assessment of the impact of disease and treatment across the physical, psychological, social and somatic domains of functioning and well-being		How satisfied are you with the current level of function of your shoulder? (PENN shoulder score)

Using the ICF framework, researchers can evaluate individual items of content, by a standardized coding, or “linking” procedure [29, 30]. According to the ICF linking rules, a second level codes start with the letters b, s, d and representing the classification of body function and structure, activity, participation, environmental factors and personal factors followed by a numeric code for the chapter number (one digit) and another two digits as the second level [29]. The linking process is a universal language that can be used to define the content of items and describe their meaningful contructs [26]. A systematic review conducted in 2013 evaluated the content of 475 shoulder related outcome measures by linking invidual items to ICF codes [18]. This work provided some evidence on content validity of shoulder PROM. We are building on this work by focusing on shoulder arthroplasty to understand the use of PROM in this area of practice, providing an updated assessment of PROM usage, and classifying the conceptual framework according to standard definitions.

The objective of the current study was to analyze the classification and content of functional and quality of life measures used to evaluate the outcome after TSA using the ICF framework by (1) identifying the PROMs used for patients after TSA; (2) mapping the content of the individual items using second level ICF codes; (3) summarizing the focus of these PROMs based on ICF domains; and (4) providing an updated assessment of PROM usage and summarizing the predominant application of included PROMs based on ICF linking and pre-defined concepts of FDH, HRQoL, and QoL.

## Methods

### Design

**Fig. 1:**
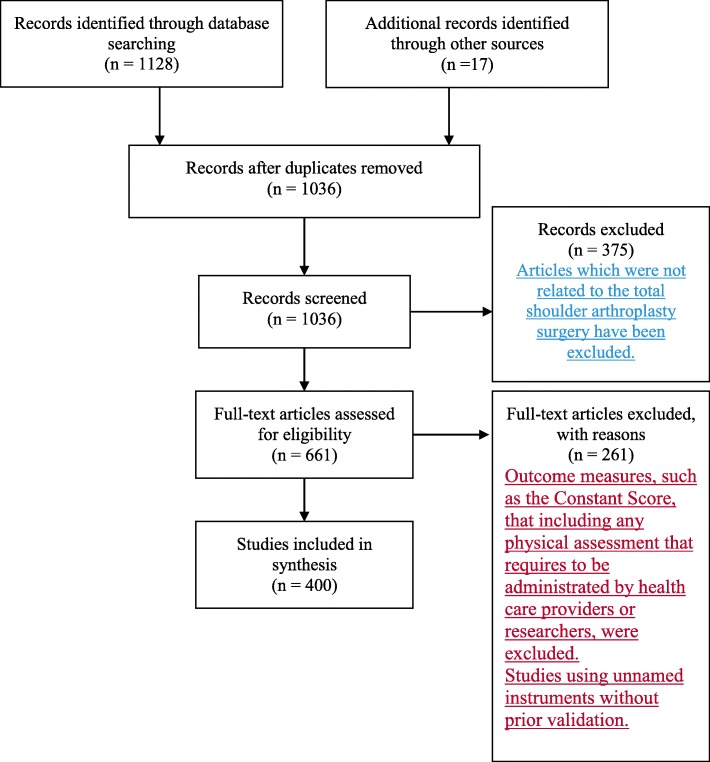
PRISMA flow diagram of the literature search with the total number of identified measures and their number of citations

A structured literature review was carried out following the PRISMA guideline [31]. The PRISMA flow diagram containing all steps of the screening and extraction of measures are displayed in Fig. 1. The content analysis of PROMs used for patients post TSA surgery was performed based on the existing ICF linking rules [29, 30].

### Information sources

A literature search was performed in three databases including MEDLINE, EMBASE, and CINAHL to capture PROMs used for patients with TSA in both clinical and research settings.

### Search

MeSH terms were used for PROMs, including questionnaire, score, index, tool, survey, outcome measure, and patient-report, were connected by Boolean operator ‘OR.’ The same operator was applied for other MeSH terms that specified TSA management by total shoulder arthroplasty and total shoulder replacement. PROMs and TSA terms were then combined with the operator ‘AND’ for final search. We limited searching to the last 5 years and 3 months, from January 2014 to December 2019, to reflect recent practice. Details of search keywords are listed in Additional file 1.

### Eligibility criteria

The inclusion criteria were any articles published in peer-reviewed journals on studies of TSA surgery using named PROMs to measure FDH, QoL or HRQoL. Outcome measures, such as the Constant Score, that including any physical assessment that requires to be administrated by health care providers or researchers, were excluded since our focus was PROM. We also excluded studies using unnamed instruments without prior validation.

### Study selection

All identified articles were imported into Mendeley reference management software (version 1.19., 2008 Glyph & Cog, LLC) for duplicate, author and journal information checking. After removal of the duplication, the first author [ZL] performed the title, abstract and full-text review. At full-text review stage, the second author [JMacD] randomly reviewed 50% of the articles and discussed the disagreement with the first author through regular meetings.

### Data collection process

Data extraction was initially performed by the first author [ZL]. The original intention of using the instrument (e.g., to measure pain, function, QoL, HRQoL, surgery outcome, patient satisfaction, etc.) was recorded. Ambiguous or difficult cases were presented through online-based discussion for the final decision. We calibrated the details of PROMs, such as different versions of the questionnaires, according to a previous systematic review and a guideline of shoulder outcomes measures [18]. To avoid detailed analysis of rarely used PROM, we excluded PROMS that had not been cited at least 10 times from the overall data pool of 1175 citations thereby excluding those that represent less than 1%The tracking sheet of excluded questionnaires is available upon request.

### Data items

According to the predetermined definitions of FDH, QoL, and HRQoL, the original intention of applying the PROMs was recorded and analyzed through the data extraction [23]. The first author documented text in the articles where they referred to researchers’ purpose of using PROMs and then coded the outcome measures for different conceptual applications. Direct clarifications in terms of function, disability or health, QoL or HRQoL were categorized onto terms FDH, QoL, or HRQoL. Ambiguous statements were coded with the consideration of the context of studies. For example, patients’ satisfaction level with their shoulder condition was coded as HRQoL.

### Summary measures (content analysis)

The content of included PROMs was evaluated item by item based on existing ICF linking rules [23, 29, 30]. One of the authors [ZL] finished the entire linking work independently and then presented the result to an external expert with experience in ICF. Any discrepancies were marked as addressed if agreement was achieved. Meaningful concepts were linked to the specific second level of the ICF codes. An individual item can map onto several codes if needed. For example, *pain pushing with the involved arm* contains meaningful concepts as *pain* and *pushing with involved arm,* which was coded separately as *sensation of pain* (b280) and *hand and arm use* (d445). General concepts that cannot be assigned with a code but are still within the classification system were linked as *non-definable* [18, 23, 29]. For example, the general evaluation of the health condition was coded as *nd* due to the coverage of all aspects of health without specific definitions. *Not covered (nc)* was used for the concepts beyond the ICF conceptual framework, such as the satisfaction level about the quality of health care Personal factor was labeled as *pf,* and consistent with ICF were acknowledged, but not coded.

We used summary indices to decide the extent to which content of a measure can be captured with ICF codes [32, 33]. The formula was listed as follows: The number of items linked to at least one ICF code/total number of items on the measure × 100%.

### Synthesis of results (from the content analysis)

Individual item codes were then categorized into the five ICF domains, including *body function and structure, activity, participation, environmental factors and personal factors* according to the linking result.

As the final step, included PROMs were summarized into FDH, HRQoL and QoL measures with the recommended use based on the previous analysis. Measures focusing on pain, shoulder function, capacity, performance, difficulty, barriers or facilitators of contextual factors were categorized as FDH measures. The dominant perspectives of measures were provided based on content analysis using ICF. Other questionnaires that mainly ask expectations, evaluation, and person judgment about health or health-related domain were coded as HRQoL. QoL measures were also classified based on the WHO definition. FDH measures with HRQoL/QoL features were given when at least one item from FDH scales was not covered by ICF component but within the HRQoL/QoL. For example, if one item from a given measure was categorized onto HRQoL related content, while other questions were all identified as FDH, this specific PROM was considered as a FDH measure with HRQoL features.

Previous evidence from the literature review was cross-referenced at this stage. Consensus was required from all three authors to finalize the result [25, 33].

## Results

### Study selection

Overall, 1036 studies were screened through the title and abstract review, and 400 of these articles were included. We identified thirty-five measures that have been cited 1175 times from all retrieved studies. Among them, five were single item questionnaires, and 30 were multi-item measures. Please see Additional file 2 for all 35 outcome measures. The Constant and three other non-PROMs, including the Constant-Murley and Charlson Morbidity Index, were not involved in further content analysis. Numeric rating scales (NRS) and visual analog scales (VAS) for pain were considered as the same measure due to similar meaning of the content of the question. The same strategy was applied for the Single Assessment Numeric Evaluation (SANE) and Subjective Shoulder Value (SSV). All studies used an English version of the PROMs. In total, 12 PROMs for our inclusion and exclusion criteria and underwent detailedr ICF linking and conceptual analysis.

### Results of individual studies (second level of ICF linking)

A total of 36 s level ICF codes were linked to individual items (Table 2). There were 23 different codes under the *activities and participation* category (*d* codes) and 10 under the *body structure (s* codes*) and body function (b* codes*)*. *Personal factors* were identified within three included PROMs: The Disabilities of the Arm, Shoulder, and Hand (DASH), Western Ontario Osteoarthritis Score (WOOS), and PENN shoulder score (PSS). Only two codes under environment factors were identified *as products or substances for personal consumption* (e110) and *climate* (e225). Eleven of the total linked codes were found with a frequency above 5% as *sensation of pain* (b280), *hand and arm use* (d445), *recreation and leisure* (d920), *remunerative employment* (d850), *lifting and carrying objects* (d430), *doing housework* (d640), *muscle power functions* (b730), *dressing* (d540), *washing oneself* (d510), *carrying out daily routine* (d230), and *sleep functions* (b134). The occasions of using these codes to link individual item in each PROMs were listed in rank order in Table 2.

**Table 2:** Second level ICF categories linked to the individual items from included PROMs in ranked order

ICF code linked to items	*b280 Sensation of pain*	*d445 Hand and arm use*	*d920 Recreation and leisure*	*d850 Remunerative employment*	*d430 Lifting and carrying objects*	*d640 Doing housework*	*b730 Muscle power functions*	*d540 Dressing*	*d510 Washing oneself*	*d230 Carrying out daily routine*	*b134 Sleep functions*
Identified PROMs (Number of items)											
ASES [17]	4	2	2	2	1		1	1	1		1
VAS&NRS-pain	1										
SST [12]	1	5		1	3		1	1	1		1
SANE & SSV [1]	***None***										
Sf-12 (12)	1		2	4		2				4	
SPADI [13]	5	3			2		1	3	2		
DASH [34]	4	7	7	5	1	5	2	1	2	1	1
WOOS [19]	4	3	1		1	1	1	2		1	1
Quick-DASH [19]	4	2	5	5	1	4	1		1	1	1
OSS [12]	4	1		1	1	2		1	1		
PSS [26]	3	5	2	1	7	1	4	3	3		1
Patient satisfaction [1]	***Not covered***										

In a total, 36 s level ICF codes were used to link individual items

Eleven of the total linked codes were found with a frequency above 5%

*ASES* American Shoulder and Elbow Society

*SST* Simple Shoulder Test

*DASH* Disabilities of the Arm, Shoulder, and Hand

*WOOS* Western Ontario Osteoarthritis Score

*PSS* PENN shoulder score

*NRS* Numerous Numeric rating scales

*VAS* Visual analog pain scales

*SANE* Single Assessment Numeric Evaluation

*SSV* Subjective Shoulder Value

*SPADI* Shoulder Pain and Disability Index

*OSS* Oxford Shoulder Scale

Of all the measures, one item proposed as “Since beginning therapy for your shoulder, would you say that your shoulder has” from PSS could not be linked by specific categories but was considered still within the ICF framework (nd). Six PROMs including SANE, SSV, SF-12, DASH, WOOS, and PSS had a question that was not covered by the ICF but was within HRQoL. One item of WOOS, asking how much of a burden do you feel you are on others, was categorized as QoL-related content. A summary of the distribution of items from each PROM under the ICF chapter level is listed by frequency order in Table 3.

**Table 3:** Categorization of items under ICF domains with corresponding percentage

	Body function and structure (25%)	Activity (41%)	Participation (24%)	Environment (2%)	Personal factors (2%)	Not defined but within ICF (1%)	QOL/HRQOL (5%)
**ASES**	2	8	4	3			
**VAS&NRSpain**	1						
**SST**	5	9	1				
**SANE & SSV**							1
**Patient satisfaction**							
**SF-12**	3	1	7				3
**SPADI**	6	12					
**DASH**	10	17	16		1		1
**WOOS**	7	7	2	1	2		3
**Quick-DASH**	6	4	12				
**OSS**	4	7	2				
**PSS**	7	18	5		1	1	2

*ASES* American Shoulder and Elbow Society

*SST* Simple Shoulder Test

*DASH* Disabilities of the Arm, Shoulder, and Hand

*WOOS* Western Ontario Osteoarthritis Score

*PSS* PENN shoulder score

*NRS* Numerous Numeric rating scales

*VAS* Visual analog pain scales

*SANE* Single Assessment Numeric Evaluation

*SSV* Subjective Shoulder Value

*SPADI* Shoulder Pain and Disability Index

*OSS* Oxford Shoulder Scale

### Synthesis of results (summarization of predominant application)

An overview of the summarized information for each PROMs is presented in Table 4. The most frequently used PROM was the American Shoulder and Elbow Society score (ASES) accounting for 21% (246 times) of the total citations, followed by the NRS or VAS for 17% and SST for 12%. Most of the analyzed measures were used as functional outcome instruments. Through the review, we found that the SF-12 was often used as a tool to evaluate QoL, although it was designed as a health status measure. Patient satisfaction scales were used to quantify the personal expectation to the surgery, care or shoulder conditions.

**Table 4:** Description of the FDH, HRQoL, and QoL perspective based on ICF linking

	Percentage of total citations	Measure to ICF linkage	Dominant ICF Component	Dominant intention	Instrument recommendations
**ASES**	21%	100%	Activity	Functional outcome	FDH instrument focusing on activity concerns
**VAS-pain**	17%	100%	Body function	Pain evaluation	FDH instrument for pain
**SST**	12%	100%	Activity Body function	Functional outcome	FDH instrument focusing on activity and body function
**SANE & SSV**	8%	0	none	Functional outcome	HRQoL
**Patient satisfaction**	5%	0	none	Patient satisfaction	Unknown
**SF-12**	3%	92%	Participation	QoL	FDH instrument focusing participation with HRQoL feature
**SPADI**	3%	100%	Activity Body function	Functional outcome	FDH instrument focusing on activity and body function
**DASH**	3%	100%	Activity Participation Body function	Functional outcome	FDH instrument with HRQoL
**WOOS**	2%	95%	Activity Body function	Functional outcome	FDH instrument focusing on activity and body function with HRQoL and QoL feature
**Quick-DASH**	2%	100%	Participation	Functional outcome	FDH instrument focusing on participation
**OSS**	2%	100%	Activity	Functional outcome	FDH instrument focusing on activity concerns
**PENN**	1%	92%	Activity	Functional outcome	FDH instrument focusing on activity with HRQoL feature

*FDH* Functioning, disability and health

*HRQoL* Health-related quality of life

*QoL* Quality of life

*ASES* American Shoulder and Elbow Society

*SST* Simple Shoulder Test

*DASH* Disabilities of the Arm, Shoulder, and Hand

*WOOS* Western Ontario Osteoarthritis Score

*PSS* PENN shoulder score

*NRS* Numerous Numeric rating scales

*VAS* Visual analog pain scales

*SANE* Single Assessment Numeric Evaluation

*SSV* Subjective Shoulder Value

*SPADI* Shoulder Pain and Disability Index

*OSS* Oxford Shoulder Scale

The high percentage of the measure to ICF linkage indicated that most of the items from included PROMs can be linked with second-level ICF, except for SANE/SSV and patient satisfaction, which are not linkable constructs. Ten of the PROMs included in this study were categorized as FDH measures, with specific focus of quantifying symptoms and functional limitations for people with shoulder problems.

## Discussion

This study found variation between commonly used PROM used to assess the outcomes of TSA in terms of their overall latent construct and the item level content, although most were more focused on activity and participation than patients perceptions of body structure and function. Overall, the content covered by the PROM included 10 s level ICF codes under the domain of *body functions and structures*, and 23 codes belong to *activities and participation*. This is consistent with the fact that PROMs focus on the patient be experience and uniquely able to assess how a person functions in their own life; whereas impairments in body structure and function can be better measured with clinical tests. Only two categories under *Environmental factors* were mentioned. Other content analysis of PROMs has noted a similar lack of attention to the environment [18]. Even where environment is not explicitly addressed, we expect it to be an important factor in disability that may partially explain why patients with similar impairments experiences different disability.

Pain is a primary concern for patients with TSA surgery [6]. This is consistent with that one category the s*ensation of pain (b280),* was ranked as the most frequently used code. Although pain is considered an impairment in ICF, it also a subjective experience and as such typically captured by PROM. The second most linked code under *body function* domain was *muscle power function* (b730), which belongs to the impairment domain under the ICF framework. Strength can be assessed by clinicians using dynamometers or other devices; or can be self-reported by patients. Typically, we expect that PROM would focus on functional items and that pain, motion and strength might all interfere with functional performance. However, some PROMs do ask questions that specifically target muscle strength. Generally, these questions must be fairly generic rather than target specific muscle groups as might be assessed by dynamometers For example, questions from SST that ask participants to rate the difficulty of lifting task with three pre-defined weight levels ranging from one lb. to 20 lbs., assess strength, but do not identify particular muscle groups or adaptations.

*Activit*y was the predominant ICF domain, accounting for 41% of the items [21, 28]. *Hand and arm use* and *lifting and carrying objects* are the most commonly linked ICF codes under the *activity* domain. This suggests a consistent recognition of the importance of these tasks in patients with shoulder arthritis, requiring TSA. However, on the other hand, the ICF categories related to mental function such as *sleep function, emotional function, and energy and drive* were infrequently linked suggesting less agreement that these are central to TSA outcomes. Given the importance of psychological health [18, 35] in post-surgical patients, one might consider this as under-representation, especially if the instrument is intended to measure QoL. However, outcome instrument developers often try to focus on a clear construct, and it would be the responsibility of researchers to include measures of physical health and psychologic health within their studies, since summing different construct together may not always be appropriate. Further, developers may consider psychological factors as mediators of outcomes rather than the outcomes themselves. Ideally, developers would be explicitly explaining these conceptual assumptions.

*Recreation and leisure* (d920) and *Remunerative employment* (d850) were ranked as third and fourth order among all the linked items. This high ranking is consistent with a previous systematic review focusing PROMs of shoulder pain and functioning [18]. Most PROMs such as ASES, DASH, and Oxford Shoulder Scale (OSS) imply these concepts by formulating questions as leisure activities and usual work. Overall, these items and others that fit within participation comprise 24% of the total items. The concept of *participation* defined by the ICF framework is subject to qualifiers that describe what a person does in their usual life. That means subjects’ response to such questions might be modified by the usual roles or environmental factors, but these are not directly measured.

According to the WHO, different PROMs used for patients after TSA share areas of content and purposes of application. The single item measure SANE, and SSV, that investigate to what extent a patient would rate their shoulder as being normal, was classified as HRQoL perspective since it address a global evaluation, whereas researchers and clinicians commonly use it as a functional outcome since it assumed to be rating physical function on a scale of 0–100% [36]. A previous study found that patients have a lot of confusion about what is being calibrated when responding to this questions, which reflect the ambiguity in its definition [37]. It is important to have a conceptual distinction between measures designed to assess HRQoL which is intended to be comprehensive, versus those designed to measure physical functioning which a smaller construct that might affect QoL. The confusion we found in conceptual clarity and content of items across many of these measures emphasizes the importance for instrument developers to define their conceptual framework so that users of outcome measures can match the measurement purpose is to a specific conceptual framework.

Patients satisfaction is important, but often variably measured in health research. Satisfaction with care is a process measure; whereas as satisfaction with health/shoulder status can be considered as an HRQoL measure. However, by asking about the satisfaction with surgery and care, this scale mixes evaluation of the process of care or Quality of Care [34], with outcome evaluation. Researchers and clinicians should be more explicit about whether they are measuring process or outcome satisfaction; and ensuring their selected measure reflects that choice.

A key issue in the literature was the vague and imprecise terminology used to for different outcome measures and the definition of the FDH, QoL, and HRQoL. For researchers, clearly defined concepts within PROMs help them detect the most appropriate and precise latent construct. For clinicians, in both research and daily practice work, appropriate selection of the outcomes measures is not only depend on the psychometric properties and intention of the application, but also on the precise understanding of the content informed by an unified conceptual framework [25]. Developers rarely provide a strong conceptual framework, and users rarely state their measurement rationale or the content validity of the tools they selected for the constructs of interest. Rather justification of PROMs within studies tend to focus on psychometric properties like reliability, which do not reflect content validity. Some measures mix different constructs. For example, The DASH provides a comprehensive set of items and is defined an as FDH instrument but contains items that fall within a HRQoL construct. Terms are often used incorrectly, for example health status measures and functional measures are often referred to as QoL measures. The use of terms like clinical outcome measures or functional outcomes happens without clear distinction about what these terms mean [11, 38]. The conceptual analysis performed in the current study may help resolve the issue by precisely categorizing the retrieved PROMs into three types as: (1) FDH (the capacity, performance, presence / absence, frequency, severity, or other biopsychosocial domains), (2) HRQoL (the expectations, standards, or concerns about individual health), and (3) QoL (the patient’s personal assessment of their position in life). Mapping the ICF domains within PROM can help researchers and clinicians to select the most appropriate PROMs for their context (and research question). That is considering the impacts of shoulder joint destruction (or indications for TSA) and expected impacts of TSA (outcomes) should drive the PROM that have the best conceptual match. Further, this can identify when important constructs are missing, and supplemental measures might be needed. This would complement, not replace, considering important psychometric properties like reliability and responsiveness.

TSA outcomes measures should be developed under a clear conceptual framework. Many of the consensus panels that attempt to achieve consensus on outcome measurement start with defining the core constructs that should be measured for a given health problem, and then choose the best measure within those constructs [25, 33, 39]. Our findings could support such a process. A better understanding of the latent construct evaluated within PROM is essential to enables clinicians and researchers to make valid conclusions. For those validated PROMs, clinician should also be cautious to use them in different conditions such as other language versions. The cross-cultural adaption might not be able to ensure the content validity with the consideration of the various culture background, healthcare systems.

### Limitation

The current review does have limitations. Our search strategy may not have identified all studies using PROMs. However, the large number of studies we reviewed created robust findings. Our exclusion of rarely used PROM may have missed some emerging but higher quality PROM that have different or more clear content validity. Extraction of data was complicated by a lack of clear reporting in some papers. Even with the updated version of ICF linking rules, personal factors are still not classified F [29], and so while we acknowledge these as important they were not classified. ICF coding is one approach to assess content validity and should be supplemented by other methods including cognitive interviews and quantitative patient/expert ratings of relevance.

## Conclusion

We found confusion in conceptual definitions on PROMs, and wide variation in PROM content and use. Despite the variability there were some common constructs evident in measurement of pain, hand and arm use, recreational activities work and employment, lifting and carrying. Mental function components such as emotional function, and energy and drive were rarely covered reflecting the focus on physical recovery following TSA. Users evaluated in these constructs may require supplemental PROM. Efforts to the consensus on the key constructs that should be measured following TSA are needed.

## Supplementary information

**Additional file 1.**

**Additional file 2.** The list of all 35 outcome measures.

## Data Availability

Not applicable.

## Abbreviations

PROMs: Patient-reported outcome measures
ICF: International Classification of Functioning, Disability and Health
ISOQOL: International society for quality of life
FDH: Functioning, disability, and health
WHO: World Health Organization
WHOQOL-BREF: WHO-Quality of Life brief version
HRQoL: Health-Related Quality of Life
QoL: Quality of Life
TSA: Total shoulder arthroplasty
ASES: American Shoulder and Elbow Society
SST: Simple Shoulder Test
DASH: Disabilities of the Arm, Shoulder, and Hand
WOOS: Western Ontario Osteoarthritis Score
PSS: PENN shoulder score
NRS: Numeric rating scales
VAS: Visual analog scales
SANE: Single Assessment Numeric Evaluation
SSV: Subjective Shoulder Value
SPADI: Shoulder Pain and Disability Index
OSS: Oxford Shoulder Scale
