# Supplementary material for: A narrative review and content analysis of functional and quality of life measures used to evaluate the outcome after TSA: an ICF linking application
Source: BMC Musculoskelet Disord. 2020 Apr 13;21:228. doi: 10.1186/s12891-020-03238-w (PMC7155280; doi:10.1186/s12891-020-03238-w)
Supplement: Supplementary file 1 — Additional file 1. [file 12891_2020_3238_MOESM1_ESM.docx]

**Ovid search**

1. Total shoulder arthroplasty.mp. or total shoulder arthroplasty/
2. total shoulder replacement.mp. or total shoulder arthroplasty/
3. Questionnaire.mp. or questionnaire/
4. score.mp.
5. index.mp.
6. tool.mp.
7. survey.mp.
8. patient-reported outcome/ or outcome measure.mp.
9. 1 or 2
10. 3 or 4 or 5 or 6 or 7 or 8
11. 9 and 10
12. limit 11 to yr="2014 -Current"

**PubMed Search**

(("arthroplasty, replacement, shoulder"[MeSH Terms] OR ("arthroplasty"[All Fields] AND "replacement"[All Fields] AND "shoulder"[All Fields]) OR "shoulder replacement arthroplasty"[All Fields] OR ("total"[All Fields] AND "shoulder"[All Fields] AND "replacement"[All Fields]) OR "total shoulder replacement"[All Fields]) OR ("arthroplasty, replacement, shoulder"[MeSH Terms] OR ("arthroplasty"[All Fields] AND "replacement"[All Fields] AND "shoulder"[All Fields]) OR "shoulder replacement arthroplasty"[All Fields] OR ("total"[All Fields] AND "shoulder"[All Fields] AND "arthroplasty"[All Fields]) OR "total shoulder arthroplasty"[All Fields])) AND (((((("surveys and questionnaires"[MeSH Terms] OR ("surveys"[All Fields] AND "questionnaires"[All Fields]) OR "surveys and questionnaires"[All Fields] OR "questionnaire"[All Fields]) OR score[All Fields]) OR ("abstracting and indexing"[MeSH Terms] OR ("abstracting"[All Fields] AND "indexing"[All Fields]) OR "abstracting and indexing"[All Fields] OR "index"[All Fields])) OR tool[All Fields]) OR survery[All Fields]) OR ("patient reported outcome measures"[MeSH Terms] OR ("patient"[All Fields] AND "reported"[All Fields] AND "outcome"[All Fields] AND "measures"[All Fields]) OR "patient reported outcome measures"[All Fields] OR ("patient"[All Fields] AND "reported"[All Fields] AND "outcome"[All Fields]) OR "patient reported outcome"[All Fields])) AND ("2014/01/01"[PDAT] : "2019/12/31"[PDAT])
