# Supplementary material for: A narrative review and content analysis of functional and quality of life measures used to evaluate the outcome after TSA: an ICF linking application
Source: BMC Musculoskelet Disord. 2020 Apr 13;21:228. doi: 10.1186/s12891-020-03238-w (PMC7155280; doi:10.1186/s12891-020-03238-w)
Supplement: Supplementary file 2 — Additional file 2. The list of all 35 outcome measures. [file 12891_2020_3238_MOESM2_ESM.docx]

| 1. ASES: American Shoulder and Elbow Society |
| --- |
| 1. VAS: Visual analog pain scales |
| 1. SST: Simple Shoulder Test |
| 1. Constant |
| 1. Patient satisfaction |
| 1. SANE: Single Assessment Numeric Evaluation |
| 1. SSV: Subjective Shoulder Value |
| 1. UCLA Shoulder Score |
| 1. 12-Item Short Form Survey (SF-12) |
| 1. SPADI: Shoulder Pain and Disability Index |
| 1. DASH: Disabilities of the Arm, Shoulder, and Hand |
| 1. Constant-murley |
| 1. WOOS: Western Ontario Osteoarthritis Score |
| 1. Quick DASH |
| 1. OSS: Oxford Shoulder Scale |
| 1. Pain |
| 1. 36-Item Short Form Survey |
| 1. PSS: PENN shoulder score |
| 1. NRS: Numeric rating scale |
| 1. EQ-5D |
| 1. shoulder activity level |
| 1. Charlson Comorbidity Index |
| 1. HADS: Hospital Anxiety and Depression Scale |
| 1. BRS: Brief Resilience Scale |
| 1. VR-12: Veterans RAND 12 Item Health Survey |
| 1. Patient-Reported Outcomes Measurement Information System |
| 1. WHOQOL-BREF |
| 1. 15D The health-related quality of life (HRQoL) |
| 1. The Pain Catastrophizing Scale |
| 1. 11-item DASH |
| 1. Korean shoulder scoring |
| 1. ADLER score: Activities of Daily Living [ADL] which require active External Rotation [ER]) |
| 1. MAC SHOULDER ACTIVITY |
| 1. Rowe Score |
| 1. WORC: Western Ontario Rotator Cuff Index |

Additional file 2 The list of all 35 outcome measures.
